# An Observational Study of SGLT2 Inhibitors and Their Use in Autosomal Dominant Tubulointerstitial Kidney Disease

**DOI:** 10.21203/rs.3.rs-7482366/v1

**Published:** 2025-09-25

**Authors:** Kendrah O. Kidd, Adrienne H. Williams, Elhussein A.E. Elhassan, Abbigail Taylor, Lauren Martin, Alice Kim, Michael V. Rocco, Michael J. Choi, Martina Zivna, Stanislav Kmoch, Peter J. Conlon, Anthony J. Bleyer

**Affiliations:** Wake Forest School University of Medicine; Wake Forest School University of Medicine; Beaumont Hospital; Wake Forest School University of Medicine; Wake Forest School University of Medicine; Wake Forest School University of Medicine; Wake Forest School University of Medicine; MedStar Georgetown University Hospital; Charles University; Charles University; Beaumont Hospital; Wake Forest School University of Medicine

**Keywords:** Autosomal Dominant Tubulointerstitial Kidney Disease, ADTKD, MUC1, UMOD, SGLT2 inhibitors, Sodium-glucose cotransporter 2 inhibitors

## Abstract

**Background:**

There are no specific treatments available for autosomal dominant tubulointerstitial kidney disease (ADTKD). As SGLT2 inhibitors have proven effective in other forms of CKD, we performed an observational study to determine their safety and effect on kidney function in ADTKD.

**Methods:**

We obtained clinical and laboratory data before and after starting an SGLT2 inhibitor on 27 individuals with ADTKD. We created a propensity-matched cohort from a Wake Forest prospective ADTKD observational study.

**Results:**

Of the 27 individuals, 12 stopped medication prior to one year due to concerns about the (anticipated) acute rise in serum creatinine. There were no adverse effects. The slope of eGFR after SGLT 2 inhibitor initiation was − 2.61 ± 2.58 ml/min/1.73m^2^, which was not significantly different from the eGFR slope prior to initiation of SGLT2 inhibitors (−3.13 ± 5.39 ml/min/1.73 m^2^ (p = 0.71)) or from 30 propensity-matched controls (−2.25 ± 2.39 ml/min/1.73 m^2^ (p = 0.83)). Hemoglobin and plasma and urine KIM-1 levels did not change after starting SGLT2 inhibitors.

**Conclusions:**

SGLT2 inhibitors were well tolerated in ADTKD patients. There was neither a rise in hemoglobin levels nor a fall in plasma or urinary KIM-1 levels, suggesting that these agents may not be beneficial in ADTKD.

## INTRODUCTION

Sodium-glucose cotransporter 2 (SGLT2) inhibitors are effective treatments for patients with diabetic nephropathy^1^ and other forms of chronic kidney disease (CKD)^2^, but there is little information available about their use in inherited disorders. It is also unclear if these agents are effective in nonproteinuric CKD.^2^

Autosomal dominant tubulointerstitial kidney disease (ADTKD) is the third most common genetic form of inherited kidney disease^3^ and is characterized by autosomal dominant inheritance, a bland urinary sediment, and CKD leading to kidney failure at a mean age of 45 years (range 20 to 80 years).^4^ The two most common forms of ADTKD are ADTKD-*UMOD* (due to *UMOD* pathogenic variants)^5^ and ADTKD-*MUC1* (due to *MUC1* pathogenic variants)^6^. While ADTKD is not associated with proteinuria, ADTKD-*UMOD* is characterized by increased tubular energy utilization due to increased proximal tubular uptake of sodium and urate.^7^ Both ADTKD-*UMOD* and ADTKD-*MUC1* have a strong component of tubulinterstitial fibrosis^8^, which could be targeted by SGLT2 inhibition.^9^ At present, there are no treatments available for ADTKD.

The purpose of this study was to follow individuals with ADTKD who were started on SGLT2 inhibitors by their healthcare providers and assess patient tolerance and change in estimated glomerular filtration rate (eGFR) over time. We also compared eGFR change with a propensity-matched cohort of participants in the Wake Forest Prospective ADTKD Cohort who were not receiving SGLT2 inhibitors.

## Materials and Methods

All participants signed informed consent to participate in the studies approved by Wake Forest University Health Sciences institutional review board and Beaumont Hospital ethics committee.

The Wake Forest Rare Inherited Kidney Disease Team conducted a prospective observational study of participants with genetically diagnosed ADTKD-*UMOD* or ADTKD-*MUC1* beginning in March 2025 and accruing 270 participants (see Table 1). Serum creatinine values were assessed at baseline and then at 3-month intervals. Plasma and urine for biomarkers were obtained at baseline and in follow up at three-month intervals and stored in a − 80°C freezer. Participants filled out a survey every three months, including questions specifically about starting SGLT2 inhibitors. As the study was observational in nature, all decisions regarding starting and stopping medications were made between the participants and their clinical providers.

All participants receiving SGLT2 inhibitors received information from their clinical providers prior to starting these agents that there would be an expected decline in eGFR after initiation of therapy, and that this eGFR decline would be reversible.^1^ Participants were told that SGLT2 inhibitors were proven beneficial in proteinuric kidney diseases^1,2^ but not in ADTKD.

Beaumont Hospital in Dublin, Ireland has a genetic kidney disease clinic and follows participants longitudinally with ADTKD.^10^ Beaumont Hospital investigators were able to identify participants started on SGLT2 inhibitors and provide baseline and follow-up eGFR values.

Statistical analysis: Baseline characteristics were calculated using standard statistical techniques with SAS (Cary, NC). In comparisons of the change in eGFR over time, participants were included if they remained on SGLT2 inhibitors for greater than 1 year. For calculation of the eGFR slope prior to initiation of SGLT2 inhibition, we used eGFR data from the prior year. The initial serum creatinine value used to calculate the slope of decline while on SGLT2 inhibition was defined as the serum creatinine value obtained at least three months after therapy initiation in order to account for the initial drop in eGFR when starting SGLT2 inhibitors.^2,11^ A propensity score was created to match participants who had taken SGLT2 inhibitors with participants not on SGLT2 inhibitors using the PSMATCH optimal algorithm in SAS with a ratio of two controls per case. Samples were matched exactly on sex and ADTKD diagnosis and with the age and eGFR using propensity scores. Four eGFR values per sample were required over a 2-year period to calculate the slope of decline of eGFR for each participant. The differences in the mean eGFR decline for individuals taking SGLT2 inhibitors and individuals not taking SGLT2 inhibitors was evaluated using a paired t-test. The differences in the mean eGFR decline prior to and after starting SGLT2 inhibitors were also evaluated using a paired t-test.

## Results

**Figure 1:**
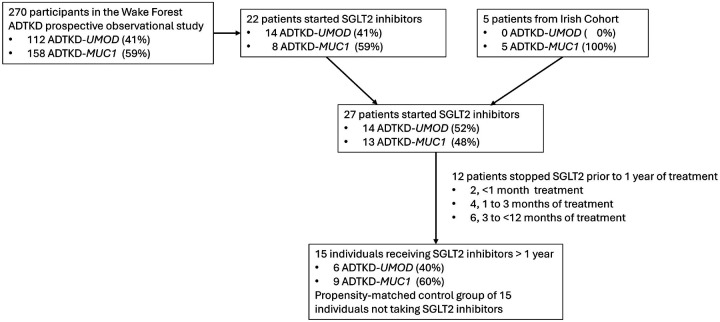
Flow diagram

There were 270 participants followed in the observational study over time (see Fig. 1 and Table 1), with 112 (41%) with ADTKD-*UMOD* and 158 (59%) with ADTKD-*MUC1*. 14 of the ADTKD-*UMOD* participants and 8 of the ADTKD-*MUC1* participants started SGLT2 inhibitors. Data was then added on 5 participants from the Irish Cohort (see Fig. 1 and Table 2).

None of the participants had underlying diabetes or other significant comorbid conditions. None of the participants had proteinuria. Thus, there were 27 individuals with ADTKD who started SGLT2 inhibitors.

Of these 27 individuals starting SGLT2 inhibitors, 2 stopped medication prior to one month, 4 stopped medication between 1 and 3 months, and 6 stopped prior to one year of treatment (Fig. 1). None of the individuals stopped SGLT2 inhibitors due to urinary tract infections, fungal infections, volume depletion, or ketoacidosis. Table 3 shows characteristics of individuals who stopped versus continued on therapy.

**Figure 2:**
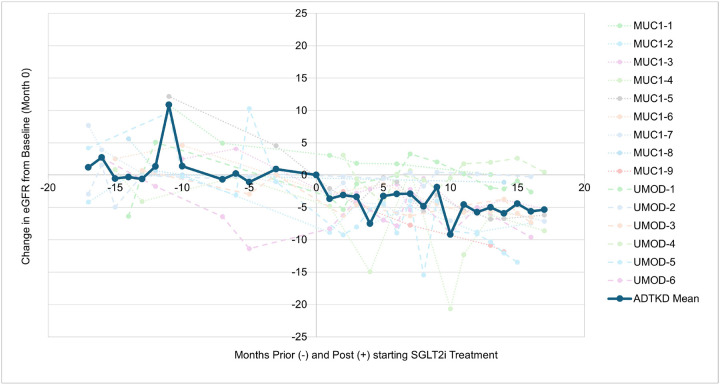
Change in eGFR over time. Mean values for each time point are only provided if there were more than 3 measurements for that time point. The eGFR decline of 4 ml/min/1.73m2 seen at 3 months was similar to prior studies of SGLT2 inhibitors.

There were 15 participants with enough data to compare longitudinal changes in eGFR. Table 4 provides characteristics of these patients, and Table 5 provides information on the propensity-matched cohort. Figure 2 shows the change in eGFR over time. Participants had a decline in eGFR at 3 months of 4 ml/min/1.73 m^2^, which was similar to the decline in eGFR observed in other studies.^1,2^ The slope of eGFR after the initiation of the SGLT2 inhibitors was − 2.61 ± 2.58 ml/min/1.73m^2^. This was not significantly different from the slope of the propensity matched controls (−2.25 ± 2.39 ml/min/1.73 m^2^ (p = 0.83)) and not significantly different from the eGFR slope prior to initiation of SGLT2 inhibitors (−3.13 ± 5.39 ml/min/1.73 m^2^ (p = 0.71)) (see Table 6). There were no differences according to ADTKD sub-type (**see** Table 7).

There was no significant change in hemoglobin values for 17 individuals who had values pre- and post-SGLT2 inhibitor initiation (see Table 8). Plasma and urinary Kim-1 values were widely dispersed. There was an increase in these values, but due to the wide standard deviation this was not statistically significant. The serum uric acid levels showed a non-significant decline.

## Discussion

SGLT2 inhibitors have been found to be an extremely effective therapy to slow the rate of eGFR decline in participants with diabetic nephropathy^1^ and proteinuric CKD.^2,12^ SGLT2 inhibitors also reduce proteinuria in Alport syndrome.^13^

It is unclear whether SGLT2 inhibitors are beneficial in non-proteinuric kidney disease^2^ or in genetic disorders autosomal dominant polycystic kidney disease.^14–16^

Participants with ADTKD suffer from tubulointerstitial kidney disease. Participants with ADTKD-*UMOD* have increased proximal tubular uptake of sodium and uric acid^17^, with increased energy expenditure of the proximal tubule, providing a potential attractive target for SGLT2 inhibitors. However, as there are a number of potential mechanisms of renal protection with SGLT2 inhibitors^18^, one cannot predict theoretically for which kidney diseases they will be effective.

There are numerous obstacles in determining if SGLT2 inhibitors will be effective in ADTKD. First, the patient population is small, and a well-powered prospective randomized trial cannot be carried out. Second, participants with ADTKD do not have proteinuria. SGLT2 inhibitors have been found to be effective in proteinuric kidney disease^2^ and also lower proteinuria^13^, a marker of efficacy. Participants with ADTKD do not have proteinuria, and there is no similar biomarker of disease activity.

Safety: SGLT2 inhibitors were well-tolerated by participants with ADTKD, with no significant adverse side effects. This is of note, as decreased uromodulin secretion in individuals with ADTKD-*UMOD* could place them at increased risk of urinary tract infections or genital infections while on SGLT2 inhibitors.^19^ There was a high dropout rate due to the expected decline in eGFR after the initiation of therapy. Participants with proteinuric kidney disease may be more likely to stay on SGLT2 inhibitor therapy after the initial eGFR decline because it is anticipated and because large prospective trials have shown that these medications will slow decline in eGFR. While participants with ADTKD were warned that they would have a decline in eGFR, many participants were uncomfortable with this decline, mostly because there was no long-term assurance of benefit as in proteinuric kidney disease trials. There were no significant differences in eGFR decline or other characteristics for participants who stayed on vs. stopped therapy.

The initial change in eGFR mirrored that of other SGLT2 inhibitor studies. We also assessed two other endpoints associated with SGLT2 inhibitor efficacy. First, a rise in hemoglobin has been associated with the efficacy of SGLT2 inhibitors. In an analysis by Wanner and colleagues, the 12 week change in hematocrit from baseline was the strongest mediator of beneficial outcomes (99.5% mediation) in participants receiving SGLT2 inhibitors.^20^ In our participants we did not find a rise in hemoglobin. Second, declines in urinary Kim-1 levels have been found in participants taking SGLT2 inhibitors^21,22^, decreasing 23% (p = 0.05) in a 6 week cross-over trial.^22^. While we have not found an association between plasma Kim-1 levels and kidney survival in our cohort, we did find a rise in Kim-1 levels in our participants. This rise was not significant due to a large standard deviation in these measurements. Increased expression of Kim-1 is associated with worse kidney outcomes in animal models.^23^

## Conclusions

In summary, we were able to show that SGLT2 inhibitors are well tolerated in participants with ADTKD, though the agents were often stopped because of the initial anticipated decline in eGFR and the uncertainty of future benefit. Hemoglobin levels did not rise, and plasma Kim-1 levels did not decline. These findings, together with findings of decreased efficacy in nonproteinuric kidney diseases^12^, were suggestive that these medications may not be effective. On the other hand, participants did have the characteristic eGFR decline with initial therapy that has been associated with future preservation of kidney function. When a biomarker of disease activity is identified in participants with ADTKD, we will reanalyze samples from this study to further look at the potential benefit of these agents.

## Figures and Tables

**Table 1:** Characteristics of participants in the Wake Forest ADTKD Cohort Observational Study who started versus did not start SGLT2 inhibitors.

	Cohort participants not starting SGLT2 inhibitors		Cohort participants starting SGLT2 inhibitors		P-value^1^	
ADTKD disease type	*UMOD*	*MUC1*	*UMOD*	*MUC1*	*UMOD*	*MUC1*
N	144	104	14	8		
Age (y)	39.30 ± 14.68	37.37 ± 12.69	40.34 ± 16.89	39.77 ± 10.98	0.82	0.51
Sex (% male)	55 (38%)	35 (34%)	7 (50%)	4 (50%)	0.34	0.37
eGFR (ml/min/1.73m^2^) at entry into the study	62.29 ± 26.61	61.34 ± 24.47	45.91 ± 17.94	57.94 ± 19.02	0.0019	0.61

1 P value comparing ADTKD subtype participants starting versus not starting SGLT2 inhibitors

**Table 2:** Characteristics of individuals with ADTKD who started SGLT2 inhibitors according to country.

	US	Ireland	Total
n	22	5	27
ADTKD-*UMOD* n (%)	14 (64%)	0	14 (52%)
ADTKD-*MUC1* n (%)	8 (36%)	5 (100%)	13 (48%)
Age (y)^1^	40.13 ±14.73	43.79 ± 20.58	40.81 ±15.57
Sex (%male)	11 (50%)	2 (40%)	13 (48%)
eGFR at entry^1^	50.29 ± 18.84	47.67 ± 19.49	49.80 ± 18.61
Dapagliflozin, n (%)	11 (50%)	5 (100%)	16 (59%)
Empagliflozin, n (%)	11 (50%)	0	11 (41%)

1 mean ± s.d.

**Table 3:** Comparison of individuals who stopped and did not stop SGLT2 inhibitors.

	Stopped SGLT 2 inhibitor < 3 months	Stopped SGLT2 inhibitor from 3 months to 1 year	Did not stop	P value
N	2	5	20	
UMOD n(%)	2 (100%)	3 (50%)	9 (45%)	0.28^1^
	0	2 (40%)	11(55%)	
Age at entry	24.58 ± 2.41	41.60 ± 24.61	42.24 ± 13.19	0.49
Age at start of SGLT2i	38.31 ±8.83	44.57 ± 24.63	50.87 ± 8.83	0.30
Sex (%Male)	0	0.40	0.55	0.27
eGFR at entry^2^	39.18 ± 3.67	41.53 ± 12.04	52.93 ± 20.06	0.42
eGFR at start of SGLT2i^2^	29.85 ± 3.94	32.24 ± 10.28	37.19 ± 8.83	0.72
% eGFR decline at one month	0.08 ± 0.11	0.08 ± 0.05	0.07 ± 0.11	0.88
Dapagliflozin, n(%)	1(50%)	4 (80%)	0.55	0.52
Empagliflozin, n(%)	1 (50%)	1 (20%)	0.45	

1 P value compares the group of individuals who stopped versus did not stop.

2 Mean ± s.d.

**Table 4:** Comparison of laboratory values

	N	Baseline	After 1–3 months (first available value)	P value from paired t test
Hemoglobin (g/dl)	17	11.99 ± 0.90	12.24 ± 1.55	0.61
Plasma Kim-1	12	897.71 ± 2083.82	961.84 ± 2269.83	0.33
Urine Kim-1	12	985.75 ± 1383.68	1682.81 ± 1221.56	0.14
Serum uric acid level	9	5.82 ± 0.94	5.06 ± 1.87	0.12

**Table 5:** Characteristics of individuals with long-term follow up

	Overall	ADTKD-*MUC1*	ADTKKD-*UMOD*	P-value
N	15	9	6	
Male, n (%)	9 (60%)	5 (56%)	4 (66%)	1.0
Age starting SGLT2 inhibitor (years)	51.59 ± 16.68	52.03 ± 18.14	50.92 ± 15.87	0.91
eGFR before start of SGLT2 inhibitors (ml/min/1.73m^2^)	34.22 ± 10.40	34.28 ± 10.12	34.13 ± 11.79	0.91
eGFR > 40 ml/min/1.73m^2^ (n, column percent)	3 (20%)	2 (22%)	1 (17%)	1.0
eGFR 30 to ≤ 40 ml/min/1.73m^2^ (n, column percent)	6 (40%)	3 (33%)	3 (50%)	
eGFR < 30 ml/min/1.73m^2^ (n, column percent) and not kidney failure	6 (40%)	4 (44%)	2 (33%)	

**Table 6:** Characteristics of individuals receiving SGLT2 inhibitors and the propensity-matched cohort.

	Cases	Matched Controls	p-value
N	15	30	
Age (years)	52.04 ± 16.76	50.38 ± 13.0	0.72
Duration of follow-up	1.35 ± 0.42	1.61 ±0.58	0.13
eGFR ml/min/1.73m^2^	31.87 ±11.13	36.69 ± 9.90	0.15
Male n (%)	9 (60%)	18 (60%)	1.0
ADTKD Type	9 MUC1 (60%)	18 MUC1 (60%)	1.0
	6 UMOD(40%)	12 UMOD (40%)	1.0

**Table 7:** Comparison in changes of eGFR (ml/min/1.73m^2^) before and after SGLT2 inhibition and between cases and the propensity-matched controls.

	Slope of eGFR over time	P-value (ref group Cases After)
Cases after SGLT2 inhibitor initiation	−2.61 ± 2.58	
Propensity-matched controls	−2.25 ± 2.39	0.83
Cases before SGLT2 inhibitor initiation	−3.13 ± 5.39	0.71

**Table 8:** Comparison of changes in eGFR (ml/min/1.73m^2^) according to ADTKD subtype.

ADTKD-*MUC1*	Slope of eGFR over time	P-value (ref group Cases After)
Cases after SGLT2 inhibitor initiation	−3.46 ± 2.83	
Propensity-matched controls	−2.67 ± 3.47	0.56
Cases before SGLT2 inhibitor initiation	−3.06 ± 2.43	0.21
ADTKD-*UMOD*	Slope of eGFR over time	P-value (ref group Cases After)
Cases after SGLT2 inhibitor initiation	−1.33 ± 1.58	
Propensity-matched controls	−1.93 ± 4.51	0.69
Cases before SGLT2 inhibitor initiation	−1.03 ± 1.90	0.36

## Data Availability

The datasets generated and analyzed during the current study are not publicly available to protect patient confidentiality. We are very happy to work with any groups interested in studying this condition and providing genetic information that can satisfy their research requests. An anonymized dataset analyzed in the study will be available from the European Genome-Phenome Archive (EGA-archive.org), with request for data access.

## Abbreviations

ADTKD: autosomal dominant tubulointerstitial kidney disease
CKD: chronic kidney disease
eGFR: estimated glomerular filtration rate
SGLT2: sodium–glucose cotransporter 2

## References

[1] Wanner C, Inzucchi SE, Lachin JM Empagliflozin and Progression of Kidney Disease in Type 2 Diabetes. N Engl J Med. 7/28/2016 2016;375(4):323–34. Not in File. 10.1056/NEJMoa1515920 [doi]. 27299675

[2] Heerspink HJL, Stefansson BV, Correa-Rotter R, Dapagliflozin in Patients with Chronic Kidney Disease. N Engl J Med Oct. 2020;8(15):1436–46. 10.1056/NEJMoa2024816. 32970396

[3] Devuyst O, Olinger E, Weber S, Autosomal dominant tubulointerstitial kidney disease. Nat Rev Dis Primers Sep. 2019;5(1):60. 10.1038/s41572-019-0109-9. 31488840

[4] Olinger E, Hofmann P, Kidd K, Clinical and genetic spectra of autosomal dominant tubulointerstitial kidney disease due to mutations in UMOD and MUC1. Kidney Int May. 2020;22. 10.1016/j.kint.2020.04.038. 32450155

[5] Hart TC, Gorry MC, Hart PS, Mutations of the UMOD gene are responsible for medullary cystic kidney disease 2 and familial juvenile hyperuricaemic nephropathy. J Med Genet. 2002;12/2002(12):882–92. Not in File. 10.1136/jmg.39.12.882 PMC1757206 12471200

[6] Kirby A, Gnirke A, Jaffe DB, Mutations causing medullary cystic kidney disease type 1 lie in a large VNTR in MUC1 missed by massively parallel sequencing. Nat Genet Mar. 2013;45(3):299–303. 10.1038/ng.2543. 23396133 PMC3901305

[7] Kidd K, Vylet’al P, Schaeffer C, Genetic and Clinical Predictors of Age of ESKD in Individuals With Autosomal Dominant Tubulointerstitial Kidney Disease Due to UMOD Mutations. Kidney Int Rep Sep. 2020;5(9):1472–85. 10.1016/j.ekir.2020.06.029. 32954071 PMC7486199

[8] Vylet’al P, Kublova M, Kalbacova M Alterations of uromodulin biology: a common denominator of the genetically heterogeneous FJHN/MCKD syndrome. Kidney Int. 9/2006. 2006;70(6):1155–1169. Not in File. doi:5001728 [pii]; 10.1038/sj.ki.5001728 [doi]. 16883323

[9] Vallon V. State-of-the-Art-Review: Mechanisms of Action of SGLT2 Inhibitors and Clinical Implications. Am J Hypertens Oct. 2024;14(11):841–52. 10.1093/ajh/hpae092. PMC11471837 39017631

[10] Cormican S, Connaughton DM, Kennedy C, Autosomal dominant tubulointerstitial kidney disease (ADTKD) in Ireland. Ren Fail Nov. 2019;41(1):832–41. 10.1080/0886022X.2019.1655452. 31509055 PMC6746258

[11] Vonesh E, Tighiouart H, Ying J, Mixed-effects models for slope-based endpoints in clinical trials of chronic kidney disease. Stat Med Sep. 2019;30(22):4218–39. 10.1002/sim.8282. 31338848

[12] Group E-KC, Herrington WG, Staplin N, Long-Term Effects of Empagliflozin in Patients with Chronic Kidney Disease. N Engl J Med Feb. 2025;20(8):777–87. 10.1056/NEJMoa2409183. PMC7616743 39453837

[13] Boeckhaus J, Gale DP, Simon J, SGLT2-Inhibition in Patients With Alport Syndrome. Kidney Int Rep Dec. 2024;9(12):3490–500. 10.1016/j.ekir.2024.09.014. 39698346 PMC11652101

[14] Eswarappa M, Madden E, Shlipak MG, Sodium-Glucose Cotransporter-2 Inhibitor Therapy and Longitudinal Changes in Kidney Function among Veterans with Autosomal Dominant Polycystic Kidney Disease. Clin J Am Soc Nephrol May. 2025;16(7):940–9. 10.2215/CJN.0000000725. PMC12262919 40377984

[15] Uchiyama K, Kamano D, Nagasaka T, Open-Label, Randomized, Controlled, Crossover Trial on the Effect of Dapagliflozin in Patients With ADPKD Receiving Tolvaptan. Kidney Int Rep Apr. 2025;10(4):1063–75. 10.1016/j.ekir.2025.01.023. 40303212 PMC12034875

[16] Nakatani S, Morioka F, Uedono H, Tsuda A, Mori K, Emoto M. Dapagliflozin administration for 1 year promoted kidney enlargement in patient with ADPKD. CEN Case Rep Aug. 2024;13(4):284–9. 10.1007/s13730-023-00840-4. 38117458 PMC11294305

[17] Bleyer AJ, Woodard AS, Shihabi Z Clinical characterization of a family with a mutation in the uromodulin (Tamm-Horsfall glycoprotein) gene. Kidney Int. 7/2003. 2003;64(1):36–42. Not in File. doi:kid081 [pii]; 10.1046/j.1523-1755.2003.00081.x [doi]. 12787393

[18] Bailey CJ, Day C, Bellary S. Renal Protection with SGLT2 Inhibitors: Effects in Acute and ChronicKidney Disease. Curr Diab Rep Jan. 2022;22(1):39–52. 10.1007/s11892-021-01442-z. 35113333 PMC8888485

[19] Liu J, Li L, Li S, Effects of SGLT2 inhibitors on UTIs and genital infections in type 2 diabetes mellitus: a systematic review and meta-analysis. Sci Rep Jun. 2017;6(1):2824. 10.1038/s41598-017-02733-w. PMC5460243 28588220

[20] Wanner C, Nangaku M, Kraus BJ, How do SGLT2 inhibitors protect the kidney? A mediation analysis of the EMPA-REG OUTCOME trial. Nephrol Dial Transplant Aug. 2024;30(9):1504–13. 10.1093/ndt/gfae032. PMC11361804 38323492

[21] Satirapoj B, Korkiatpitak P, Supasyndh O. Effect of sodium-glucose cotransporter 2 inhibitor on proximal tubular function and injury in patients with type 2 diabetes: a randomized controlled trial. Clin Kidney J Jun. 2019;12(3):326–32. 10.1093/ckj/sfy122. 31198224 PMC6543969

[22] Dekkers CCJ, Petrykiv S, Laverman GD, Cherney DZ, Gansevoort RT, Heerspink HJL. Effects of the SGLT-2 inhibitor dapagliflozin on glomerular and tubular injury markers. Diabetes Obes Metab Aug. 2018;20(8):1988–93. 10.1111/dom.13301. 29573529 PMC6055757

[23] Humphreys BD, Xu F, Sabbisetti V, Chronic epithelial kidney injury molecule-1 expression causes murine kidney fibrosis. J Clin Invest Sep. 2013;123(9):4023–35. 10.1172/JCI45361. 23979159 PMC3755983

